# Numerical Study of Gas Flow in Super Nanoporous Materials Using the Direct Simulation Monte-Carlo Method

**DOI:** 10.3390/mi14010139

**Published:** 2023-01-04

**Authors:** Vahid Shariati, Ehsan Roohi, Amin Ebrahimi

**Affiliations:** 1 High-Performance Computing (HPC) Laboratory, Department of Mechanical Engineering, Ferdowsi University of Mashhad, Mashhad 91775-1111, Iran; 2 State Key Laboratory for Strength and Vibration of Mechanical Structures, International left for Applied Mechanics (ICAM), School of Aerospace Engineering, Xi’an Jiaotong University (XJTU), Xianning West Road, Beilin District, Xi’an 710049, China; 3 Department of Materials Science and Engineering, Faculty of Mechanical, Maritime and Materials Engineering, Delft University of Technology, Mekelweg 2, 2628 CD Delft, The Netherlands

**Keywords:** direct simulation Monte Carlo (DSMC), super nanoporous (mesoporous) materials, rarefied gas flow, thermal boundary conditions, hydraulic tortuosity, permeability

## Abstract

The direct simulation Monte Carlo (DSMC) method, which is a probabilistic particle-based gas kinetic simulation approach, is employed in the present work to describe the physics of rarefied gas flow in super nanoporous materials (also known as mesoporous). The simulations are performed for different material porosities (0.5≤ϕ≤0.9), Knudsen numbers (0.05≤Kn≤1.0), and thermal boundary conditions (constant wall temperature and constant wall heat flux) at an inlet-to-outlet pressure ratio of 2. The present computational model captures the structure of heat and fluid flow in porous materials with various pore morphologies under rarefied gas flow regime and is applied to evaluate hydraulic tortuosity, permeability, and skin friction factor of gas (argon) flow in super nanoporous materials. The skin friction factors and permeabilities obtained from the present DSMC simulations are compared with the theoretical and numerical models available in the literature. The results show that the ratio of apparent to intrinsic permeability, hydraulic tortuosity, and skin friction factor increase with decreasing the material porosity. The hydraulic tortuosity and skin friction factor decrease with increasing the Knudsen number, leading to an increase in the apparent permeability. The results also show that the skin friction factor and apparent permeability increase with increasing the wall heat flux at a specific Knudsen number.

## 1. Introduction

Gas flow in microporous and super nanoporous (also known as mesoporous) materials with pore sizes ranging between 100μm and 10nm [1] has recently attracted considerable attention because of its vast prospective industrial applications such as gas storage and sensing, mass separation, batteries, catalysis, and optics. The performance in many of these applications depends critically on the characteristics of transport phenomena (i.e., heat and mass transfer) through internal pore space [2,3,4]. Therefore, enhancing our understanding of the multiscale transport phenomena in porous media is required to support the development of such micro- and nanodevices [5].

At standard pressure and temperature conditions, gas flow in microporous and mesoporous materials differs considerably from those in macroporous material with pore sizes larger than about 100μm [6,7,8,9,10]. Internal pore spacing in microporous and mesoporous materials is comparable to the mean free path of gas molecules [11], enhancing nonequilibrium effects due to the reduction in the rate of intermolecular collisions and collisions between the gas molecules and solid walls [12,13,14]. The Knudsen number (Kn) is a dimensionless quantity that indicates deviation from the equilibrium condition and reads as the ratio of the molecular mean free path λ to a characteristic length scale L (i.e., Kn=λ/L). Four different gas flow regimes are customarily defined based on the value of the Knudsen number: the continuum regime with Kn<0.001, the slip regime with 0.001<Kn<0.1, the transition regime with 0.1<Kn<10 and the free-molecular regime with Kn>10 [15]. It should be noted that such a classification is intrinsically empirical, and the values of the limits of the various regimes might be different for fluid flows in complex geometries [16]. Gas flow in microporous and mesoporous materials is often in the slip and transition regimes at standard pressure and temperature [17,18], for which the continuum assumption is invalid. The standard Navier-Stokes-Fourier (NSF) equations fail to describe gas flows in porous media when the nonequilibrium effects are significant [5]. Accordingly, Darcy’s law, which is often employed to describe laminar fluid flow in a porous medium at low Reynolds numbers in the continuum flow regime, also fails to describe fluid flow in microporous and mesoporous materials [19]. The direct simulation Monte-Carlo (DSMC) method [20] is a probabilistic particle-based numerical technique based on the kinetic theory that can approximate the solution of the Boltzmann equation that governs gas flow in the entire range of Knudsen numbers.

Gas flow in microporous and mesoporous materials is characterized by extremely low permeability in the order of 10^−21^ m^2^ [11]. Moreover, the intrinsic permeability ($K_{\mathrm{int}}$) is lower than the apparent permeability ($K_{\mathrm{app}}$) in the rarefied flow regime, and the difference between these two permeabilities increases with a decrease in average gas pressure in the porous material [21]. This is because the intrinsic permeability depends on the morphology of the porous structure, whereas the gas pressure has also been accounted for in the definition of apparent gas permeability [22]. Determining the value of permeability through experimental studies is often challenging because the signal-to-noise ratio is considerably low [23]. Various theoretical models have been developed to approximate the value of permeability in microporous and mesoporous materials (see, for instance, [24,25,26,27,28,29,30,31,32]). Zhao et al. [7] employed a computational model based on the lattice Boltzmann method to simulate gas flow in digital rock models and confirmed that the apparent permeability is higher than the intrinsic permeability in porous materials, and argued that the difference between apparent and intrinsic permeabilities increases with an increase in the Knudsen number. Wang and Pan [33] replicated a porous medium using a quartet structure generation set (QSGS) model and studied the effect of specific surface area and porosity on the permeability of porous media using the lattice Boltzmann method. However, anisotropy and multiscale pore size distribution which are critical features in studying transport phenomena in porous media are neglected when using the QSGS model to replicate the porous medium geometry [34,35,36]. Further investigations are needed to enhance our understanding of the effects of influencing parameters such as gas rarefaction and thermal boundary conditions on the gas flow behavior in super nanoporous materials.

In the present work, the geometry of the porous media in super nanoporous materials is replicated using the PoreSpy library in Python. The Direct Simulation Monte Carlo (DSMC) method is employed to simulate rarefied gas flow through super nanoporous materials over a wide range of Knudsen numbers in the slip and transition flow regimes. The effects of critical influencing parameters such as material porosity, Knudsen number, and thermal boundary conditions on hydraulic tortuosity, skin friction, and permeability are discussed.

## 2. Methods

**Figure 1:**
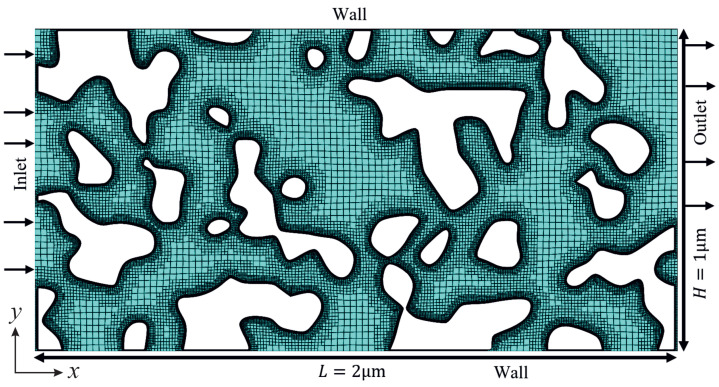
A representative geometry of the porous medium generated using the PoreSpy code, the computational grid used and the boundary conditions applied to the outer boundaries of the computational domain. Regions shaded in green indicate the fluid domain. The computational grid was generated using the SnappyHexMesh mesh generation tool.

An open-source code, PoreSpy [37], was used to replicate the geometry of the porous material for numerical simulations. PoreSpy includes a variety of predefined functions to extract data from images of porous material (for example, those obtained using X-ray tomography) and to generate artificial geometries of porous materials [37,38,39]. The Blobs function from the PoreSpy code generates an image with random noise and then applies a Gaussian blur to the image, creating a correlated field with a Gaussian distribution. Nanoporous materials often have random bicontinuous structures with complex pore morphologies in which heat and fluid flow are inherently three-dimensional. Computational costs associated with running three-dimensional models to simulate gas flow in nanoporous materials using particle-based methods, such as molecular dynamics (MD) and direct simulation Monte-Carlo (DSMC), are often considerably high and thus current studies are limited, to a great extent, to two-dimensional problems (see for instance [9,19,40,41,42]). Figure 1 shows a representative geometry of the computational domain, the grid used in the simulations, and the boundary conditions applied to the outer boundaries of the computational domain. The computational domain is a rectangular block that encompasses the porous structure and has a length (*L*) of 2μm and a height (*H*) of 1μm. Minimum cell spacing was selected to be smaller than the molecular mean free path and about λ/3 [13], resulting in a computational grid with 80,000 cells and the minimum cell spacing of about 5 × 10^−9^ for the case with a Knudsen number of 0.5. The minimum cell spacing chosen in the present simulations (Δx=Δy<λ/3) is sufficiently fine to achieve grid-independent results according to the guidelines [43,44,45]. The inlet-to-outlet pressure ratio ($P_{i}/P_{o}$) was set to 2 in the simulations.

The direct simulation Monte-Carlo (DSMC) method [20], which is a probabilistic particle-based method, was used to simulate heat and gas flow through the porous material. The DSMC method relies on decoupling molecular motion from binary intermolecular collisions. To avoid prohibitive computational costs, each simulator particle in the DSMC method represents numerous molecules in the flow field. In the DSMC method, the computational domain is discretized into cells smaller than the molecular mean free path, and motion of molecules and their collisions are computed at each time-step that is smaller than the mean collision time [20]. The simulations were constructed using an open-source DSMC solver, dsmcFoam+ [46], within the framework of OpenFOAM. The working fluid studied in the present work is argon with a molecular diameter (*d*_m_) of 4.17 × 10^−10^ m and a molecular mass (*m*_m_) of 6.63 × 10^−26^ kg. It is worth noting that the present computational model is equally applicable to other gas species. The variable hard sphere (VHS) model [20] was employed to simulate intermolecular collisions, and the no-time-counter (NTC) scheme [20] was chosen for the collision partner selection model. In the present simulations, each computational cell contained at least 20 simulator particles (i.e., PPC = 20), and the time-step size was chosen to be about one-third of the mean collision time and in the order of 10^−12^ s [3,13]. Although surface adsorption and diffusion can contribute to gas transport in super nanoporous materials [47,48,49,50], these aspects are neglected in the present DSMC simulations. All the solid walls were assumed to be fully diffuse, and heat transfer to the solid porous structure was ignored [51,52]. Detail of the present DSMC model [3,53] and the validity of the results are explained thoroughly in our previous works [3,12,13,14,19,54].

To construct a framework for presenting the results obtained from the simulations, the Reynolds number (Re), intrinsic permeability ($K_{\mathrm{int}}$), apparent permeability ($K_{\mathrm{app}}$), and hydraulic tortuosity factor ($T_{f}$) are defined as follows [3,55,56]:

(1) Re=ρUL*μ=πγ2MaKn,

(2) $$K_{\mathrm{int}}=\frac{-\mu \;U_{\mathrm{avg}}}{P_{i}-P_{o}},$$

(3) $$K_{\mathrm{app}}=\frac{2\;\mu \;L\;P_{o}\;U_{o}}{P_{i}^{2}-P_{o}^{2}},$$

(4) $$T_{f}=\frac{U_{\mathrm{avg}}}{U_{d}},$$

where, ρ is the fluid density, *U* the gas velocity, μ the dynamic viscosity, γ the specific heat ratio of the gas, Ma the Mach number, $U_{\mathrm{avg}}$ the average velocity magnitude, *L* the length of the porous structure, and $U_{d}$ the stream-wise gas velocity along the pressure gradient direction within the porous material. The subscripts ‘i’ and ‘o’ indicate inlet and outlet respectively. $L^{*}$ in Equation (1) is the characteristic length scale of the porous medium and is determined as follows:

(5) $$L^{*}=\sqrt{\frac{12K_{\mathrm{int}}}{\phi }},$$

where ϕ is the material porosity. The intrinsic permeability ($K_{\mathrm{int}}$), which is a property of the porous medium alone, is a quantity that assesses how readily a porous medium can transport a fluid under a potential gradient [57]. This means that the value of the intrinsic permeability depends solely on the porous structure, regardless of fluid condition. However, the apparent permeability ($K_{\mathrm{app}}$) of a porous medium is a measure of gas transport that comprises pressure gradient-driven flow, concentration gradient-driven molecular diffusion, and chemical gradient-driven surface diffusion [21,50].

## 3. Results and Discussion

### 3.1. Model Validation

**Figure 2:**
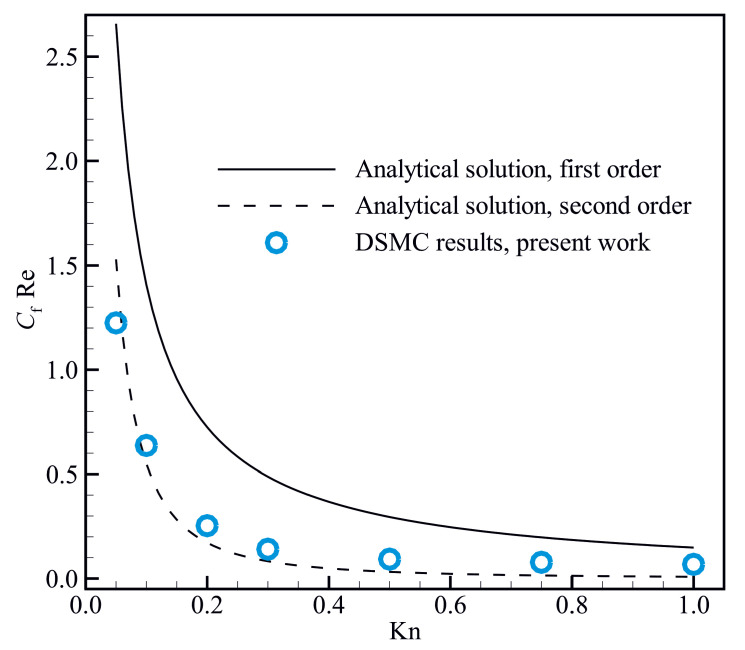
Comparison between the Poiseuille number obtained from the present DSMC simulations and the analytical approximations calculated using Equations (7) and (8).

Pressure drop of fluid flow in a channel is often evaluated using the skin friction coefficient ($C_{f}$), which represents the ratio of the skin shear stress ($\tau_{w}$) to the dynamic pressure of the free stream, and is defined as follows:

(6) $$C_{f}=\frac{2\;\cdot \;\tau_{w}}{\rho_{m}U_{\mathrm{avg}}^{2}},$$

where, $\rho_{m}$ and $U_{\mathrm{avg}}$ are the average density and the average velocity magnitude respectively. The skin friction coefficients obtained from the present DSMC simulations for isothermal steady micro- and nanoscale flows are compared with those approximated using the analytical slip models in Figure 2, indicating the validity of the present DSMC results. According to the Cercignani’s model, the Poiseuille number (CfRe) can be approximated using the first- and second-order slip coefficients as follows respectively [58]:

(7) CfRe=241+6Kn,

(8) CfRe=241+6Kn+3.72Kn2.

The accuracy of the present DSMC model is also assessed in our previous works by comparing the numerical predictions obtained from the model with experimental and theoretical data [3,12,13,14,19,54].

### 3.2. The Effects of Material Porosity on the Gas Flow Behaviour

**Figure 3:**
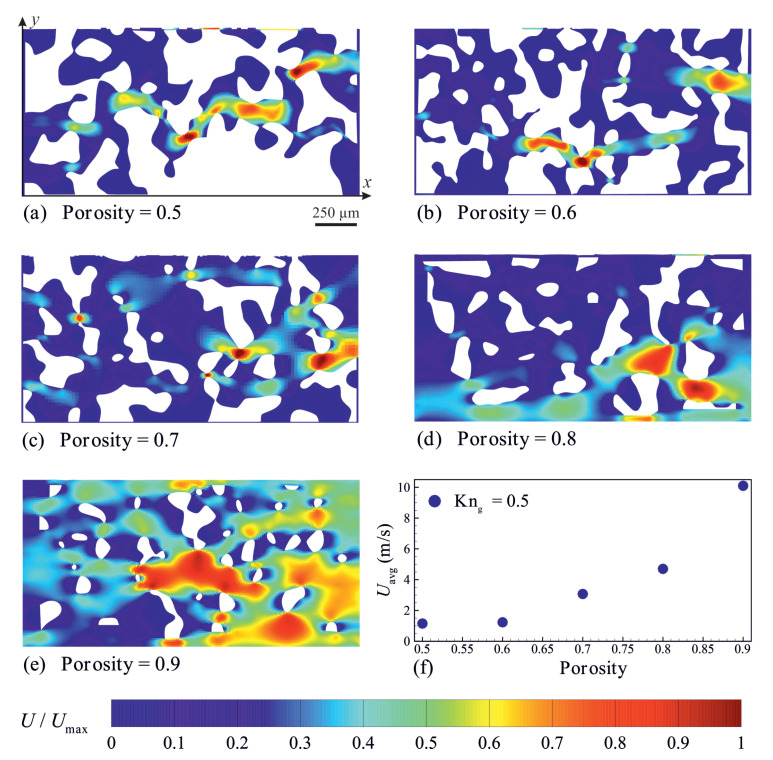
Velocity field distribution (**a**–**e**) and the predicted average velocity (**f**) in porous materials with different porosities. The velocity magnitudes (*U*) are normalised using the respective maximum velocity magnitude ($U_{\max}$). ${\mathrm{Kn}}_{g}=0.5$, $P_{i}/P_{o}=2$ and $T_{w}=300K$.

The effects of material porosity and rarefaction on the gas flow behavior in super nanoporous materials are studied for isothermal wall boundary conditions. Temperature of the solid walls *T*w is set to 300 K, unless stated otherwise. The material porosity (ϕ) is defined as the ratio of the volume of the material’s void spaces to its total volume [59]. Changes in material porosity affect the value of the local Knudsen number (${\mathrm{Kn}}_{l}$) because the characteristic length (L) based on which the Knudsen number is defined changes. Moreover, changes in material porosity can affect pore size distribution and hydraulic tortuosity factor. In the present work, the material porosity (ϕ) ranges between 0.5 and 0.9, and the Knudsen number is defined based on the channel height (i.e., ${\mathrm{Kn}}_{g}=\lambda /H$). Figure 3 shows the numerically predicted velocity field in the porous material for different material porosities. The results indicate that the shape and structure of porous material led to notable fluid flow disruption and can significantly affect the fluid flow field. The average gas velocity decreases with decreasing the material porosity ϕ. It appears that variations in the average fluid velocity become insignificant for material porosities smaller than 0.6.

**Figure 4:**
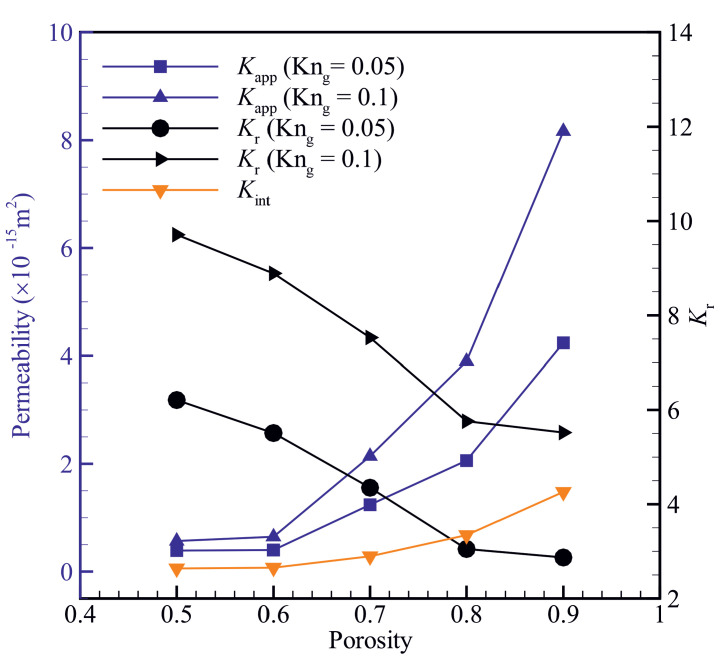
Variation of apparent permeability ($K_{\mathrm{app}}$), intrinsic permeability ($K_{\mathrm{int}}$) and the ratio of apparent to intrinsic permeability ($K_{r}=K_{\mathrm{app}}/K_{\mathrm{int}}$) with material porosity ϕ.

Figure 4 shows the variation of apparent permeability ($K_{\mathrm{app}}$) and intrinsic permeability ($K_{\mathrm{int}}$) as a function of material porosity ϕ. The apparent permeability is higher than intrinsic permeability for all the porosities studied in the present work. Both apparent and intrinsic permeabilities increase with increasing the material porosity. The ratio of apparent to intrinsic permeability ($K_{r}=K_{\mathrm{app}}/K_{\mathrm{int}}$) decreases with an increase in the material porosity. For materials with relatively high porosities, the equivalent pore size is much larger than the molecular mean free path; thus, the number of intermolecular collisions is more than the number of collisions between gas molecules and solid walls [19], enhancing the effects of viscous forces. The equivalent pore size decreases with decreasing the material porosity and can become comparable to the molecular mean free path, enhancing the effects of Knudsen diffusion.

**Figure 5:**
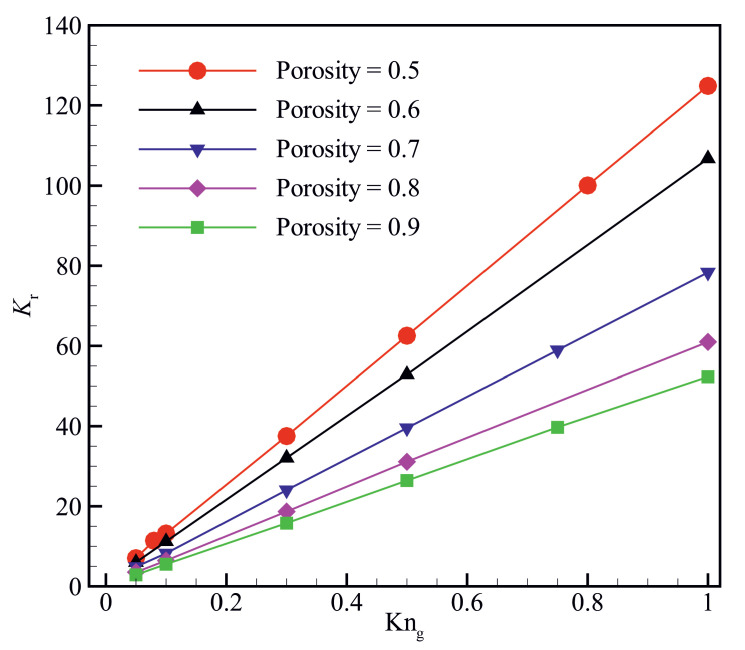
Variation of the ratio of apparent to intrinsic permeability ($K_{r}=K_{\mathrm{app}}/K_{\mathrm{int}}$) as a function of Knudsen number ${\mathrm{Kn}}_{g}$ for different material porosities.

Figure 5 shows the variation of permeability ratio $K_{r}$ with Knudsen number ${\mathrm{Kn}}_{g}$ for different material porosities. The permeability ratio $K_{r}$ increases with increasing the Knudsen number for all the material porosities studied in the present work. The higher the material porosity, the lower the permeability ratio for a specific Knudsen number. The effective characteristic length decreases with decreasing the material porosity at a specific Knudsen number (${\mathrm{Kn}}_{g}$), resulting in an increase in the local Knudsen number and hence enhancing the slippage effect on the solid walls. The local Knudsen number increases with increasing ${\mathrm{Kn}}_{g}$ at constant material porosity, enhancing the slippage effect on the solid walls. Enhancement of the slippage effect leads to an increase in the permeability ratio $K_{r}$.

**Figure 6:**
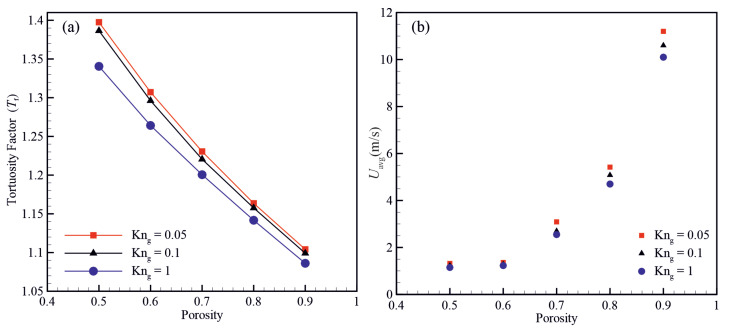
Variation of (**a**) the hydraulic tortuosity factor ($T_{f}$) and (**b**) average velocity as a function of material porosity and Knudsen number.

Figure 6 shows the effects of material porosity and Knudsen number on the hydraulic tortuosity factor ($T_{f}$) and average velocity ($U_{\mathrm{avg}}$). The average velocity field along the pressure gradient direction increases with increasing the material porosity. Moreover, the flow passage becomes more tortuous with decreasing the material porosity. The results show that the hydraulic tortuosity factor decreases with increasing the material porosity and decreases with increasing the Knudsen number.

**Figure 7:**
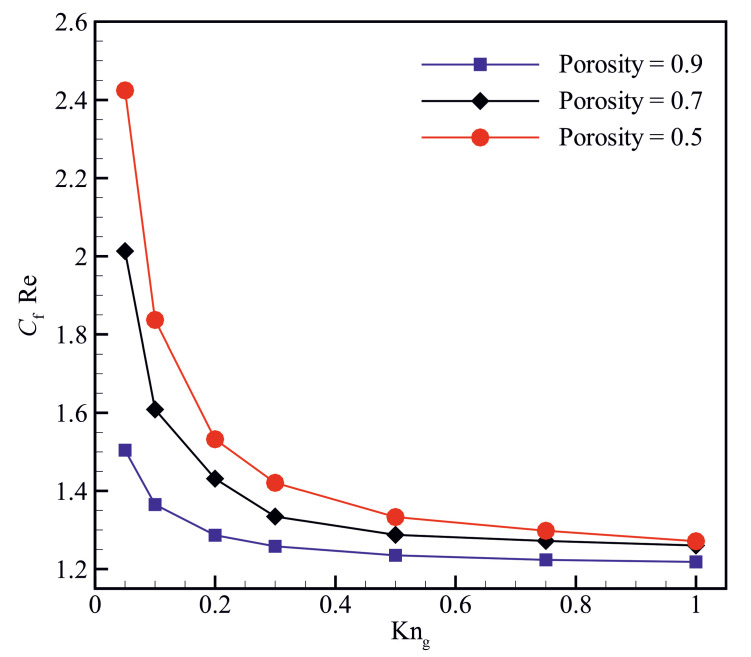
Variation of the Poiseuille number (CfRe) for different material porosities and Knudsen numbers.

Figure 7 shows the variations of the Poiseuille number (CfRe) for different material porosities and Knudsen numbers. The results indicate that the Poiseuille number increases with decreasing the material porosity at a specific Knudsen number. The Poiseuille number seems to be more sensitive to variations of the Knudsen number at low material porosity, which can be attributed to the enhancement of the collisions between gas molecules and solid boundaries at low material porosities. The Poiseuille number decreases with increasing the Knudsen number for all the material porosities studied in the present work; however, it seems that the decrease in the Poiseuille number becomes negligible for Knudsen numbers (${\mathrm{Kn}}_{g}$) larger than 0.5.

**Figure 8:**
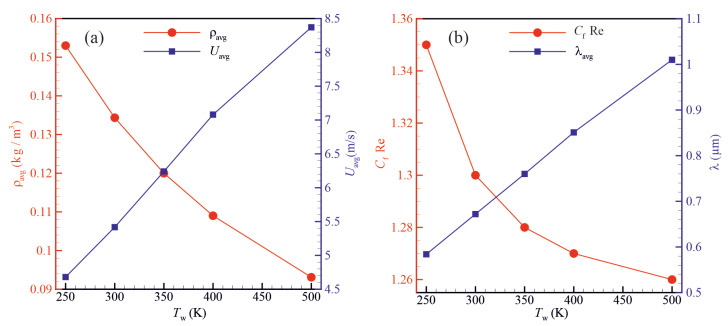
The influence of the solid wall temperature on (**a**) the density and average gas velocity and (**b**) the Poiseuille number. (${\mathrm{Kn}}_{g}=0.5$, ϕ=0.8, and $T_{i}=300\;K$).

Figure 8 shows the effect of solid wall temperature on the gas density, average fluid velocity, Poiseuille number and molecular mean free path. The density of the gas and the Poiseuille number decrease with increasing the wall temperature. Moreover, the molecular mean free path and average fluid velocity increase with increasing the solid wall temperature.

### 3.3. The Effects of Wall Heat-Flux on the Flow Behaviour

**Figure 9:**
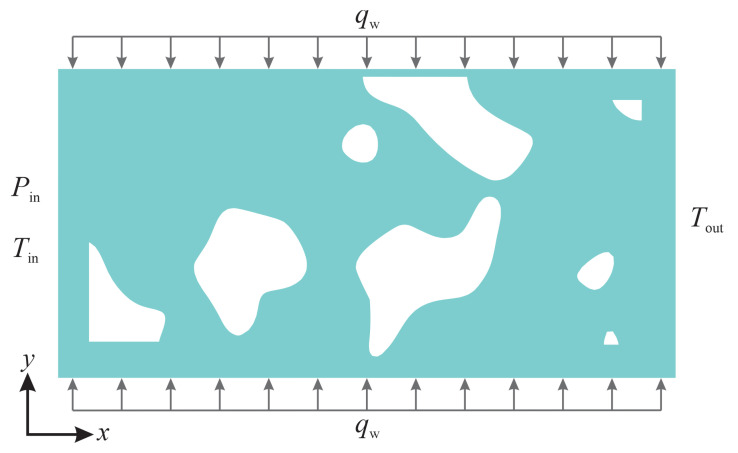
The structure of the super nanoporous material subject to constant wall heat flux boundary condition. Regions shaded in green indicate the fluid domain.

Heat and fluid flow in a super nanoporous material subject to constant wall heat-flux boundary conditions is described in this section. The material has a porosity of ϕ=0.8 and a constant wall heat-flux boundary condition is imposed on its top and bottom surfaces, as shown in Figure 9.

**Figure 10:**
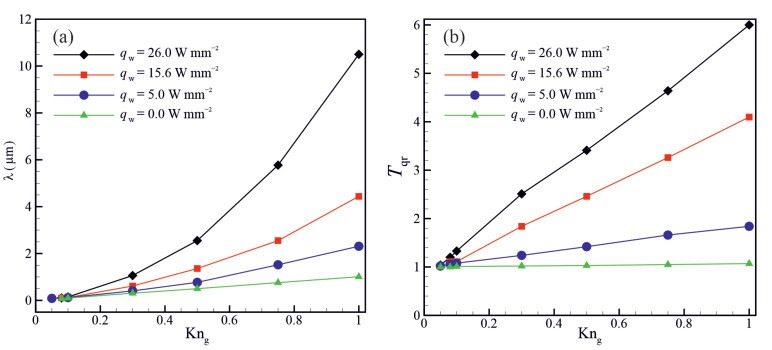
Variation of (**a**) the molecular mean free path λ and (**b**) mean bulk gas temperature as a function of Knudsen number for different wall heat fluxes.

**Figure 11:**
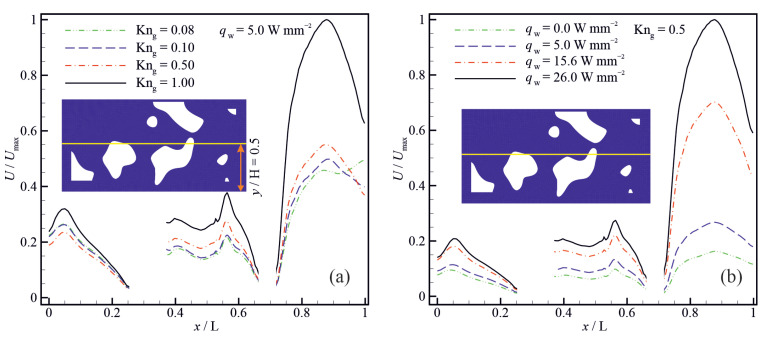
Velocity profiles along the horizontal centreline for (**a**) different Knudsen numbers and (**b**) different wall heat fluxes.

Figure 10 shows the variation of the molecular mean free path λ and the dimensionless average bulk gas temperature ($T_{\mathrm{qr}}=\bar{T_{\mathrm{gas}}}⁄\bar{T_{\mathrm{adiabatic}}}$) for different wall heat fluxes ($q_{w}$). The gas bulk temperature increases with increasing the wall heat flux, increasing in the molecular mean free path because its value is proportional to the square root of gas bulk temperature in the variable hard sphere (VHS) model [60]. The enhancement of the molecular mean free path due to increasing the wall heat flux is more pronounced at high Knudsen numbers. An increase in the bulk gas temperature, resulting from increasing the wall heat flux, also leads to a decrease in the effective gas viscosity [13,14]. An increase in the molecular mean free path, while the geometry of the porous structure is unchanged, leads to an increase in the Knudsen number, enhancing the non-equilibrium effects such as gas slippage and temperature jump at the solid walls. Hence, interactions between gas molecules and solid walls become significant compared to intermolecular collisions in transferring information by the gas flow in super nanoporous materials. Figure 11 shows the velocity profiles along the horizontal centreline for different Knudsen numbers and wall heat fluxes. The results indicate that the normalized gas velocity increases with increasing the Knudsen number and wall heat flux. The velocity profile is considerably affected by the porous structure. Due to the presence of solid particles in the flow domain, the width of the flow passage expands and contracts along the porous material. Changes in the width of the flow passage affect the gas velocity. The gas accelerates passing through the regions where the flow passage is contracted and decelerates through the regions where the flow passage expands.

**Figure 12:**
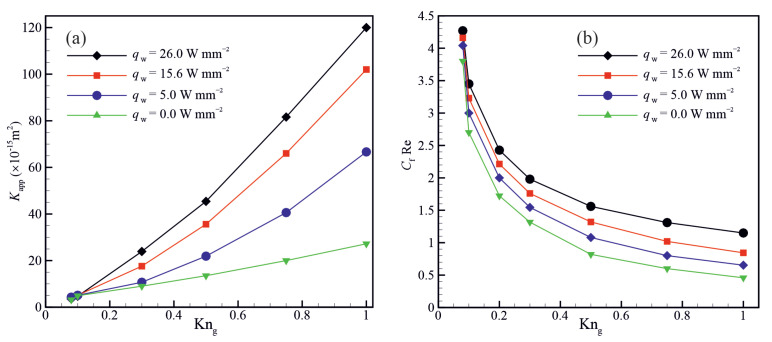
Variation of (**a**) the apparent permeability ($K_{\mathrm{app}}$) and (**b**) Poiseuille number (CfRe) as a function of Knudsen number for different wall heat fluxes.

Figure 12 shows the variation of the apparent permeability ($K_{\mathrm{app}}$) and Poiseuille number (CfRe) as a function of Knudsen number for different wall heat fluxes. The results shown in Figure 12a indicates that the apparent permeability increases with an increase in the wall heat flux. This increase in the apparent permeability can be attributed to the enhancement of the molecular mean free path and hence the rarefaction effects. Figure 12b indicates that the Poiseuille number decreases with increasing the Knudsen number and increases with increasing the wall heat flux. This is because the gas viscosity decreases with increasing the gas bulk temperature and Kundsen number.

**Figure 13:**
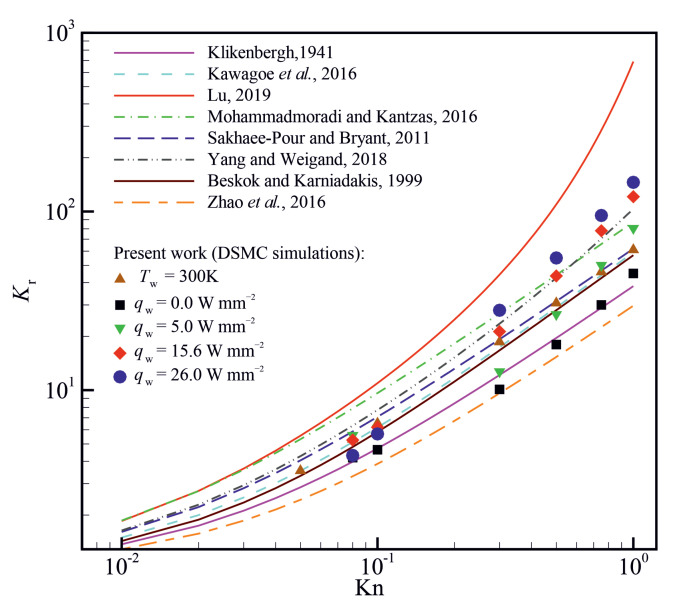
Comparison of the permeability ratio ($K_{r}$) obtained from the present DSMC simulations with available models.

Various models are proposed in the literature to approximate the value of apparent to intrinsic permeability ratio $K_{r}=K_{\mathrm{app}}/K_{\mathrm{int}}$ for rarefied gas flows, as summarised in Table 1. To the best of the authors’ knowledge, there is no reliable experimental data in the literature reporting gas flow in super nanoporous materials. In such situations, a common practice is to use analytical models and previous numerical data to assess the validity of a computational model. The permeability ratios obtained from the present DSMC simulations are compared with the models presented in Table 1, and the results are shown in Figure 13. The present DSMC results are obtained for argon flow through a porous material with a porosity of ϕ=0.8 and an inlet-to-outlet pressure ratio ($P_{i}/P_{o}$) of 2. It seems that the present DSMC results obtained using a constant wall temperature boundary condition agree reasonably with the model proposed by Kawagoe et al. [51] for a wide range of Knudsen numbers varying between $10^{-2}$ and 1. However, the model of Kawagoe et al. [51] fails to approximate the value of the permeability ratio when a constant heat flux is applied on the outer boundaries of the porous material. When an adiabatic wall boundary condition (i.e., $q_{w}=0$) is used, the DSMC results seem to agree with the model proposed by Klinkenberg [21]. However, it appears that none of the models can approximate the value of the permeability ratio with reasonable accuracy when a finite heat flux is applied to the outer boundaries of the porous material.

## 4. Conclusions

Gas (argon) flow in super nanoporous materials subjected to different thermal boundary conditions (constant wall temperature and constant wall heat flux) was studied using the direct simulation Monte-Carlo (DSMC) method. The porous structure of the material was replicated using PoreSpy, which is an open-source Python code, and the simulations were constructed using the dsmcFoam+ solver within the framework of OpenFOAM. The effects of material porosity and Knudsen number on the hydraulic tortuosity, permeability, and skin friction factor were described quantitatively. The following conclusions are drawn based on the results presented in the paper.

- The ratio of apparent to intrinsic permeability, hydraulic tortuosity, and skin friction factor increase with decreasing the material porosity.
- The hydraulic tortuosity and skin friction factor decrease with increasing the Knudsen number, leading to an increase in the apparent permeability.
- The skin friction factor and apparent permeability increase with increasing the wall heat flux at a specific Knudsen number.
- When the outer boundaries of the porous material are subjected to a constant wall temperature boundary condition, the permeability values approximated using the model proposed by Kawagoe et al. [51] agree with DSMC results for a wide range of Knudsen numbers varying between $10^{-2}$ and 1. However, the model of Kawagoe et al. [51] fails to approximate the value of the permeability ratio when a constant heat flux is applied on the outer boundaries of the porous material.
- Further investigations are required to improve the accuracy of models in approximating permeability in porous materials subject to a wall heat flux boundary condition.

A critical limitation of the DSMC method is currently related to the high computational costs, particularly for simulations of gas flows in the continuum and slip flow regimes. Further research is needed to reduce computational costs associated with DSMC simulations to make this approach a viable tool for predicting complex three-dimensional thermal and fluid flow fields in porous materials. Moreover, future studies on characterizing gas flow in microporous and super nanoporous materials could focus on developing accurate and reliable experimental measurement techniques to provide more data for validating theoretical models.

## Figures and Tables

**Table 1:** A summary of various models reported in the literature to approximate the value of permeability ratio $K_{r}$.

Reference	$K_{r}=K_{\mathrm{app}}/K_{\mathrm{int}}$	Remark
Zhao et al. [7]	1+4αKn	α=0.8
Klinkenberg [21]	1+4αKn	α=1.037
Mohammadmoradi and Kantzas [6]	1+αKn	α=9.62
Sakhaee-Pour and Bryant [61]	$1+\frac{64}{3\pi }\mathrm{Kn}$	-
Beskok and Karniadakis [62]	$\left(1+\alpha (\mathrm{Kn})\mathrm{Kn}\right)\left(1+\frac{4\mathrm{Kn}}{1+\mathrm{Kn}}\right)$	$\alpha \left(\mathrm{Kn}\right)=\frac{128}{15\pi^{2}}\arctan\left(4{\mathrm{Kn}}^{0.4}\right)$
Kawagoe et al. [51]	$1+\frac{64}{3\pi }\frac{1+c_{1}^{k}p}{1+c_{2}^{k}p}$	$c_{1}^{k}p=\sqrt{\frac{\pi }{2}}\frac{2}{\mathrm{Kn}}$ and $c_{2}^{k}p=\sqrt{\frac{\pi }{2}}\frac{2.47}{\mathrm{Kn}}$
Yang and Weigand [52]	$1+\alpha \mathrm{Kn}+\beta {\mathrm{Kn}}^{2}$	α=7.08 and β=0.485
Lu [63]	$1+8\left(3.12\exp\left(\alpha \mathrm{Kn}\right)-3.12\right)$	α=0.374

## Data Availability

The data generated in this study are available on reasonable request from the corresponding author.

## References

[1] Mays T.J. (2007). A new classification of pore sizes. Studies in Surface Science and Catalysis.

[2] Kärger J. (2014). Transport Phenomena in Nanoporous Materials. ChemPhysChem.

[3] Shariati V., Ahmadian M.H., Roohi E. (2019). Direct Simulation Monte Carlo investigation of fluid characteristics and gas transport in porous microchannels. Sci. Rep..

[4] Strizhenov E.M., Chugaev S.S., Men’shchikov I.E., Shkolin A.V., Zherdev A.A. (2021). Heat and Mass Transfer in an Adsorbed Natural Gas Storage System Filled with Monolithic Carbon Adsorbent during Circulating Gas Charging. Nanomaterials.

[5] Kalarakis A.N., Michalis V.K., Skouras E.D., Burganos V.N. (2012). Mesoscopic Simulation of Rarefied Flow in Narrow Channels and Porous Media. Transp. Porous Media.

[6] Mohammadmoradi P., Kantzas A. (2016). Pore-scale permeability calculation using CFD and DSMC techniques. J. Pet. Sci. Eng..

[7] Zhao J., Yao J., Zhang M., Zhang L., Yang Y., Sun H., An S., Li A. (2016). Study of Gas Flow Characteristics in Tight Porous Media with a Microscale Lattice Boltzmann Model. Sci. Rep..

[8] Borner A., Panerai F., Mansour N.N. (2017). High temperature permeability of fibrous materials using direct simulation Monte Carlo. Int. J. Heat Mass Transf..

[9] Gu Q., Ho M.T., Zhang Y. (2021). Computational methods for pore-scale simulation of rarefied gas flow. Comput. Fluids.

[10] Lai B., Wang Z., Wang H., Bai J., Li W., Ming P. (2022). Prediction of the permeability of fibrous porous structures under the full flow regimes. Phys. Fluids.

[11] Monteiro P.J.M., Rycroft C.H., Barenblatt G.I. (2012). A mathematical model of fluid and gas flow in nanoporous media. Proc. Natl. Acad. Sci. USA.

[12] Ebrahimi A., Roohi E. (2016). Flow and thermal fields investigation in divergent micro/Nano channels. J. Therm. Eng..

[13] Ebrahimi A., Roohi E. (2017). DSMC investigation of rarefied gas flow through diverging micro- and nanochannels. Microfluid. Nanofluid..

[14] Ebrahimi A., Shahabi V., Roohi E. (2021). Pressure-Driven Nitrogen Flow in Divergent Microchannels with Isothermal Walls. Appl. Sci..

[15] Sone Y. (2007). Molecular Gas Dynamics.

[16] Song W., Liu H., Wang W., Zhao J., Sun H., Wang D., Li Y., Yao J. (2018). Gas flow regimes judgement in nanoporous media by digital core analysis. Open Phys..

[17] Kazmouz S.J., Giusti A., Mastorakos E. (2016). Numerical simulation of shale gas flow in three-dimensional fractured porous media. J. Unconv. Oil Gas Resour..

[18] Javadpour F., Singh H., Rabbani A., Babaei M., Enayati S. (2021). Gas Flow Models of Shale: A Review. Energy Fuels.

[19] Ahmadian M.H., Roohi E., Teymourtash A., Stefanov S. (2019). A dusty gas model-direct simulation Monte Carlo algorithm to simulate flow in micro-porous media. Phys. Fluids.

[20] Bird G.A. (1994). Molecular Gas Dynamics and the Direct Simulation of Gas Flows.

[21] Klinkenberg L.J. (1941). The permeability of porous media to liquids and gases. Am. Petrol. Inst. Drill. Prod. Pract..

[22] Ghassemi A., Pak A. (2011). Pore scale study of permeability and tortuosity for flow through particulate media using Lattice Boltzmann method. Int. J. Numer. Anal. Methods Geomech..

[23] Wang J., Chen L., Kang Q., Rahman S.S. (2016). Apparent permeability prediction of organic shale with generalized lattice Boltzmann model considering surface diffusion effect. Fuel.

[24] Ziarani A.S., Aguilera R. (2011). Knudsen’s Permeability Correction for Tight Porous Media. Transp. Porous Media.

[25] Ma J., Sanchez J.P., Wu K., Couples G.D., Jiang Z. (2014). A pore network model for simulating non-ideal gas flow in micro- and nano-porous materials. Fuel.

[26] Hooman K., Tamayol A., Dahari M., Safaei M.R., Togun H., Sadri R. (2014). A theoretical model to predict gas permeability for slip flow through a porous medium. Appl. Therm. Eng..

[27] Lv Q., Wang E., Liu X., Wang S. (2014). Determining the intrinsic permeability of tight porous media based on bivelocity hydrodynetics. Microfluid. Nanofluid..

[28] Yuan Y., Doonechaly N.G., Rahman S. (2015). An Analytical Model of Apparent Gas Permeability for Tight Porous Media. Transp. Porous Media.

[29] Wu L., Ho M.T., Germanou L., Gu X.J., Liu C., Xu K., Zhang Y. (2017). On the apparent permeability of porous media in rarefied gas flows. J. Fluid Mech..

[30] Wang S., Shi J., Wang K., Sun Z., Miao Y., Hou C. (2018). Apparent permeability model for gas transport in shale reservoirs with nano-scale porous media. J. Nat. Gas Sci. Eng..

[31] Wang F., Jiao L., Lian P., Zeng J. (2019). Apparent gas permeability, intrinsic permeability and liquid permeability of fractal porous media: Carbonate rock study with experiments and mathematical modelling. J. Pet. Sci. Eng..

[32] Sabet S., Barisik M., Mobedi M., Beskok A. (2019). An extended Kozeny-Carman-Klinkenberg model for gas permeability in micro/nano-porous media. Phys. Fluids.

[33] Wang M., Pan N. (2007). Numerical analyses of effective dielectric constant of multiphase microporous media. J. Appl. Phys..

[34] Pant L.M., Huang H., Secanell M., Larter S., Mitra S.K. (2015). Multi scale characterization of coal structure for mass transport. Fuel.

[35] Yu H., Chen J., Zhu Y., Wang F., Wu H. (2017). Multiscale transport mechanism of shale gas in micro/nano-pores. Int. J. Heat Mass Transf..

[36] Tian J., Qi C., Sun Y., Yaseen Z.M., Pham B.T. (2020). Permeability prediction of porous media using a combination of computational fluid dynamics and hybrid machine learning methods. Eng. Comput..

[37] Gostick J., Khan Z., Tranter T., Kok M., Agnaou M., Sadeghi M., Jervis R. (2019). PoreSpy: A Python Toolkit for Quantitative Analysis of Porous Media Images. J. Open Source Softw..

[38] Qin C.Z., van Brummelen H., Hefny M., Zhao J. (2021). Image-based modeling of spontaneous imbibition in porous media by a dynamic pore network model. Adv. Water Resour..

[39] Wieland R., Ukawa C., Joschko M., Krolczyk A., Fritsch G., Hildebrandt T.B., Schmidt O., Filser J., Jimenez J.J. (2021). Use of deep learning for structural analysis of computer tomography images of soil samples. R. Soc. Open Sci..

[40] Zhao J., Yao J., Li A., Zhang M., Zhang L., Yang Y., Sun H. (2016). Simulation of microscale gas flow in heterogeneous porous media based on the lattice Boltzmann method. J. Appl. Phys..

[41] Wang J., Kang Q., Wang Y., Pawar R., Rahman S.S. (2017). Simulation of gas flow in micro-porous media with the regularized lattice Boltzmann method. Fuel.

[42] Li J., Ho M.T., Borg M.K., Cai C., Li Z.H., Zhang Y. (2021). Pore-scale gas flow simulations by the DSBGK and DVM methods. Comput. Fluids.

[43] Oran E.S., Oh C.K., Cybyk B.Z. (1998). Direct Simulation Monte Carlo: Recent Advances and Applications. Annu. Rev. Fluid Mech..

[44] Sun Z.X., Tang Z., He Y.L., Tao W.Q. (2011). Proper cell dimension and number of particles per cell for DSMC. Comput. Fluids.

[45] Alexander F.J., Garcia A.L., Alder B.J. (1998). Cell size dependence of transport coefficients in stochastic particle algorithms. Phys. Fluids.

[46] White C., Borg M., Scanlon T., Longshaw S., John B., Emerson D., Reese J. (2018). dsmcFoam+: An OpenFOAM based direct simulation Monte Carlo solver. Comput. Phys. Commun..

[47] Bhatia S.K., Bonilla M.R., Nicholson D. (2011). Molecular transport in nanopores: A theoretical perspective. Phys. Chem. Chem. Phys..

[48] Huang N., Chen X., Krishna R., Jiang D. (2015). Two-Dimensional Covalent Organic Frameworks for Carbon Dioxide Capture through Channel-Wall Functionalization. Angew. Chem..

[49] Liu L., Nicholson D., Bhatia S.K. (2017). Exceptionally high performance of charged carbon nanotube arrays for CO_2_ separation from flue gas. Carbon.

[50] Li W., Wang D., Wang J.G. (2022). Improved mathematical model of apparent permeability: A focused study on free and multilayer adsorptive phase flow. J. Nat. Gas Sci. Eng..

[51] Kawagoe Y., Oshima T., Tomarikawa K., Tokumasu T., Koido T., Yonemura S. (2016). A study on pressure-driven gas transport in porous media: From nanoscale to microscale. Microfluid. Nanofluid..

[52] Yang G., Weigand B. (2018). Investigation of the Klinkenberg effect in a micro/nanoporous medium by direct simulation Monte Carlo method. Phys. Rev. Fluids.

[53] Balaj M., Roohi E., Akhlaghi H., Myong R.S. (2014). Investigation of convective heat transfer through constant wall heat flux micro/nano channels using DSMC. Int. J. Heat Mass Transf..

[54] Varade V., Duryodhan V.S., Agrawal A., Pradeep A.M., Ebrahimi A., Roohi E. (2015). Low Mach number slip flow through diverging microchannel. Comput. Fluids.

[55] Germanou L., Ho M.T., Zhang Y., Wu L. (2018). Intrinsic and apparent gas permeability of heterogeneous and anisotropic ultra-tight porous media. J. Nat. Gas Sci. Eng..

[56] Jambunathan R., Levin D.A., Borner A., Ferguson J.C., Panerai F. (2019). Prediction of gas transport properties through fibrous carbon preform microstructures using Direct Simulation Monte Carlo. Int. J. Heat Mass Transf..

[57] Lohman S.W. (1972). Definitions of Selected Ground-Water Terms, Revisions and Conceptual Refinements.

[58] Hadjiconstantinou N.G. (2003). Comment on Cercignani’s second-order slip coefficient. Phys. Fluids.

[59] White W.B. (2012). Hydrogeology of Karst Aquifers. Encyclopedia of Caves.

[60] Bird G.A. (1983). Definition of mean free path for real gases. Phys. Fluids.

[61] Sakhaee-Pour A., Bryant S.L. (2012). Gas Permeability of Shale. SPE Reserv. Eval. Eng..

[62] Beskok A., Karniadakis G.E. (1999). Report: A Model for Flows in Channels, Pipes, and Ducts at Micro and Nano Scales. Microscale Thermophys. Eng..

[63] Lu Y. (2019). Higher-order Knudsen’s permeability correction model for rarefied gas in micro-scale channels. Nat. Gas Ind. B.

